# Pulmonary artery pressure as a method for assessing hydration status in an anuric hemodialysis patient – a case report

**DOI:** 10.1186/s12882-020-01924-4

**Published:** 2020-07-11

**Authors:** Anne Rudbeck Juhl, Jesper Juul Larsen, Kasper Rossing, Lisbet Brandi

**Affiliations:** 1 Department of Endocrinology and Nephrology, North Zealand University Hospital, Dyrehavevej 29, 3400 Hilleroed, Denmark; 2 Department of Cardiology, Rigshospitalet University Hospital, Blegdamsvej 9, 2100 Copenhagen, Denmark

**Keywords:** ESRD, Hemodialysis, Pulmonary hypertension, Pulmonary artery hypertension, CardioMEMS, Dry weight, Bioelectrical impedance analysis, Case report

## Abstract

**Background:**

Setting the dry weight and maintaining fluid balance is still a difficult challenge in dialysis patients. Overhydration is common and associated with increased cardiac morbidity and mortality. Pulmonary hypertension is associated with volume overload in end-stage renal dysfunction patients. Thus, monitoring pulmonary pressure by a CardioMEMS device could potentially be of guidance to physicians in the difficult task of assessing fluid overload in hemodialysis patients.

**Case presentation:**

61-year old male with known congestive heart failure deteriorated over 3 months’ time from a state with congestive heart failure and diuresis to a state of chronic kidney disease and anuria. He began a thrice/week in-hospital hemodialysis regime. As he already had implanted a CardioMEMS device due to his heart condition, we were able to monitor invasive pulmonary artery pressure during the course of dialysis sessions. To compare, we estimated overhydration by both bioimpedance and clinical assessment. Pulmonary artery pressure correlated closely with fluid drainage during dialysis and inter-dialytic weight gain. The patient reached prescribed dry weight but remained pulmonary hypertensive by definition. During two episodes of intradialytic systemic hypotension, the patient still had pulmonary hypertension by current definition.

**Conclusion:**

This case report observes a close correlation between pulmonary artery pressure and fluid overload in a limited amount of observations. In this case we found pulmonary artery pressure to be more sensitive towards fluid overload than bioimpedance. The patient remained pulmonary hypertensive both as he reached prescribed dry weight and experienced intradialytic hypotensive symptoms. Monitoring pulmonary artery pressure via CardioMEMS could hold great potential as a real-time guidance for fluid balance during hemodialysis, though adjusted cut-off values for pulmonary pressure for anuric patients may be needed. Further studies are needed to confirm the findings of this case report and the applicability of pulmonary pressure in assessing optimal fluid balance.

## Background

Still today, maintaining fluid balance in anuric patients presents a clinical challenge and nephrologists requests a valid method for assessing hydration status. Currently, clinical estimation of dry weight remains the superior method, thus standard of care in assessing hydration status [1]. More exact means of estimating hydration status is of importance, as overhydration is common in hemodialysis patients and associated with increased cardiac morbidity such as systemic and pulmonary hypertension, left ventricular hypertrophy and increased mortality [2–4]. Volume overload itself has been found as an independent risk factor for mortality, even when adjusting for blood pressure [4].

Literature reports of a connection between fluid overload and pulmonary hypertension: Pulmonary hypertension (PH) has been associated with volume overload in end-stage renal dysfunctions (ESRD) patients [4]. The PEPPER-trial investigated pulmonary hypertension in hemodialysis patients by right heart catherization (RHC) [5]. They found a higher prevalence of PH in the dialysis cohort (77%) versus the non-dialysis cohort (71%) and the cause of PH to depend mainly on fluid overload [5]. Pulmonary hypertension is currently defined as mean pulmonary artery pressure (mPAP) ≥ 20 mmHg combined with an elevated vascular resistance of ≥3 mmHg*liter*min^− 1^ (revised in 2019, previously defined at mPAP ≥25 mmHg at rest measured by RHC) [6].

Though the PEPPER-trial’s use of RHC was breaking at the time, it has the limitation of only evaluating pulmonary artery pressure (PAP) before and after dialysis, with no option of monitoring pressure during dialysis. However, this has become possible by a CardioMEMS device, primarily used in the field of cardiology: A pressure sensor implanted into the distal branch of the descending pulmonary artery in a procedure like RHC. Daily pressure readings can be done with home equipment, lasting 10 s per reading. A physician can then immediately access the pressure reading by a secure database. Besides the measured value of the pulmonary pressure, a waveform for the 10 s reading is available for qualitative assessment [7].

It seems obvious from the connection between PH and overhydration that it is of interest to put pulmonary pressure to the test as a method for guidance of fluid status during hemodialysis. The CardioMEMS device holds the potential to be a clinical applicable real-time marker of hydration status during dialysis; however, this is yet to be investigated.

Here we present a case of an anuric hemodialysis patient with an implemented CardioMEMS device, allowing us to invasively monitor pulmonary pressure during the course of hemodialysis.

## Case presentation

61-year-old man was referred to the cardiologist at a highly specialized Danish university hospital, Rigshospitalet, for an evaluation of severe congestive heart failure (CHF) in 2017. He had a history of CHF, dilated cardiomyopati since many years and left ventricular ejection fraction at 20%. About half a year ago, he had an ablation due to tendency to ventricular tachycardia. He was assessed to NYHA class III and had multiple heart failure-related hospitalizations in the past few years. He presented a clinical challenge, as it was difficult to control his fluid balance. He was deemed a candidate for a CardioMEMS device, which was implanted in the fall 2018 without complications. His pulmonary artery pressure was found too high, and subsequently tried lowered by many different kinds of diuretics. However, trying to drain his fluid overload by diuretics turned out to have severe adverse effects:

- ○ Kidney parameters increased rapidly
- ○ Hypokalemia
- ○ Ventricular tachycardia

**Fig. 1:**
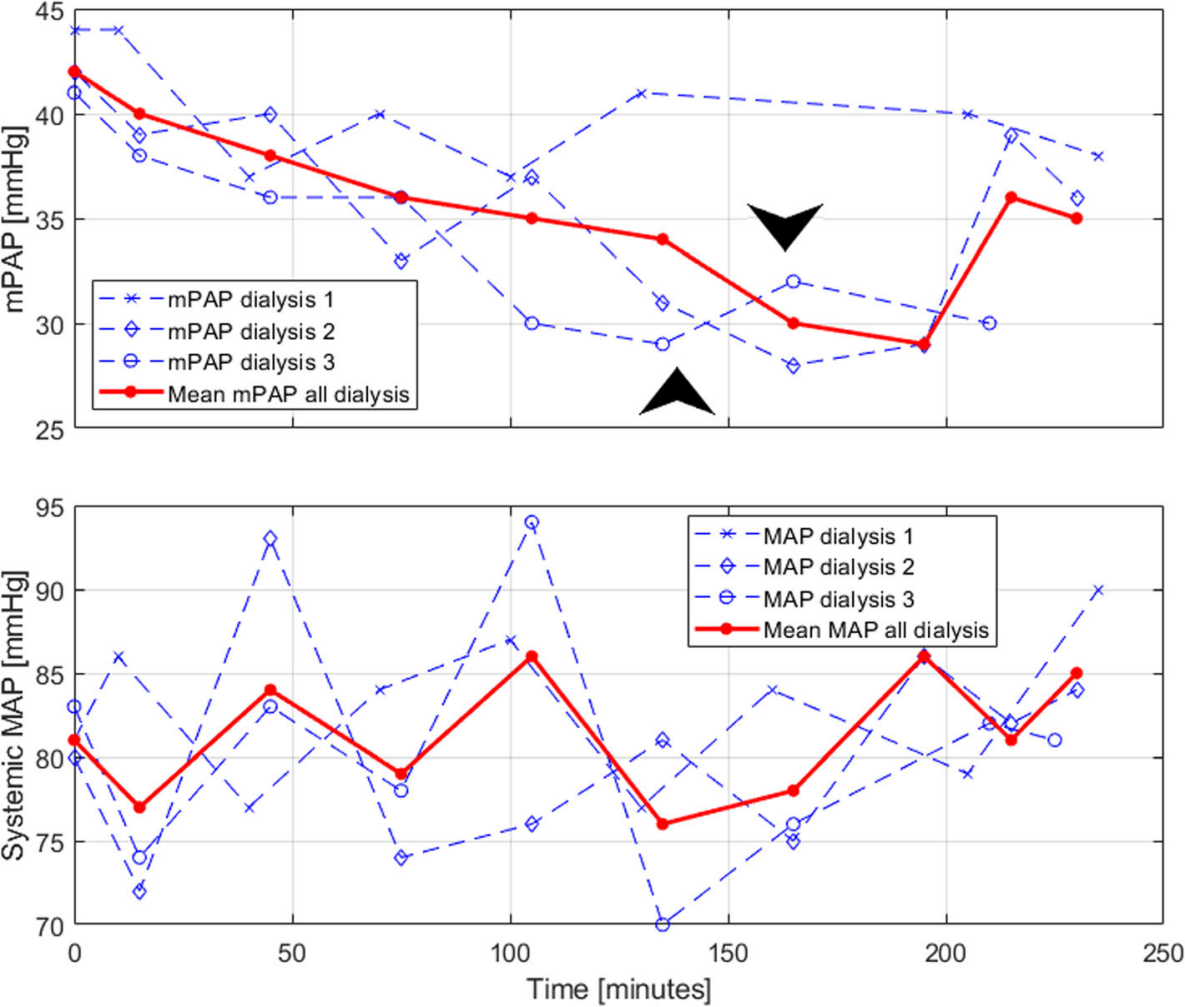
Mean pulmonary artery pressure (mPAP) and mean arterial systemic blood pressure (MAP) as a function of time during hemodialysis. The arrows marks events of hypotension during dialysis session 3 leading the dialysis nurse to pause ultrafiltration for 5 min per event

**Fig. 2:**
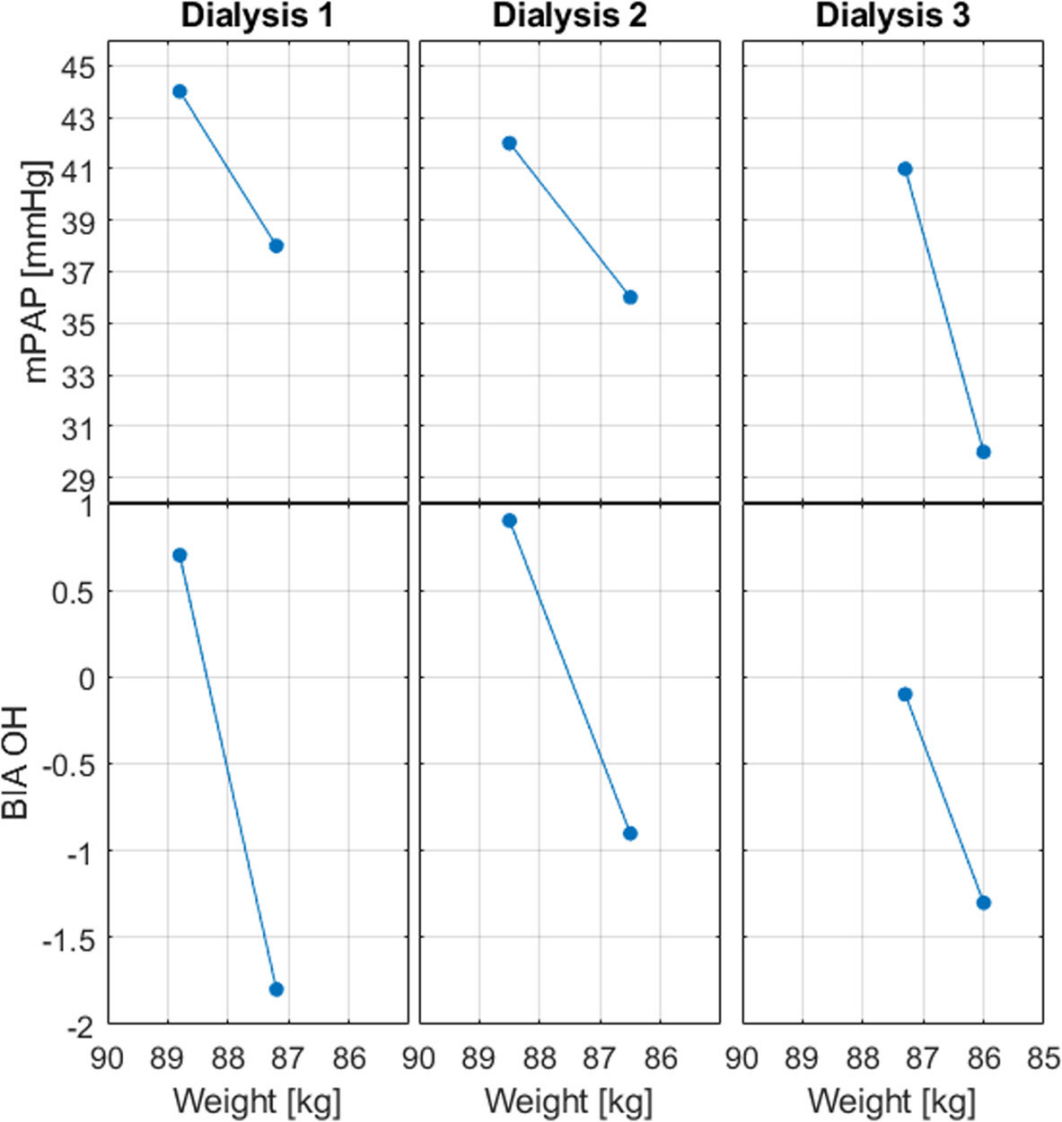
Mean pulmonary artery pressure (mPAP) and BIA-assessed overhydration (OH) as a function of weight

In a little more than 3 months’ time, the patient went from a state of CHF with diuresis to a state with chronic kidney disease and anuria. He started on a hemodialysis regime with in-hospital dialysis three times weekly through a central venous catheter. After 8 dialysis sessions, he was in start 2019 referred to Department of Nephrology at a bigger university hospital outside Copenhagen, North Zealand University Hospital, Hillerød. This rare combination of having an implanted CardioMEMS device and ongoing hemodialysis gave the possibility to invasively monitor pulmonary pressure in course of the dialysis sessions, and compare the results to clinical assessment and bioimpedance analysis. The sessions were conducted as follows in Table 1. Results are shown in Figs. 1 and 2. Bioimpedance (BIA) was measured by a multifrequency portable whole-body bioimpedance spectroscopy device (Fresenius Medical care). By intradialytic hypotension is understood a decrease in systemic blood pressure along with clinical symptoms as assessed by the responsible dialysis nurse. Pre-dialytic blood samples was planned once weekly. Endpoint was set to be either 10 dialysis, patient death or withdrawal for other reasons.

**Table 1:** Conduction and data of dialysis sessions

		Data	Remarks
Conduction PAP/MAP		PAP-readings made at intervals: - Before beginning of dialysis - After 15 min of dialysis - Once every 30 min from that point - At the end of dialysis - 15 min after ended dialysis MAP measured at the same intervals	Mean pulmonary artery pressure (mPAP) and mean systemic artery pressure (MAP) is used for discussion in this case report
Dialysis data	Session 1	Mean UF-rate: 743 ml/hr UF-volume: 2590 ml Hemoglobin: 6.0 mmol/L Abumin: 35 g/L Urea: 24.4 mmol/L Creatinine: 204 μmol/L Alkaline phosphatase: 115 U/L Pro-BNP: 1120 pmol/L	A total of one PAP reading has been omitted due to a technical error Blood samples planned for session 1, 4, 7, 10.
	Session 2	Mean UF-rate: 800 ml/hr UF-volume: 2770 ml	
	Session 3	Mean UF-rate: 524 ml/hr UF volume: 1600 ml	Final post-dialytic reading has not been obtained due to patient’s compliance, as he was not admitted and wished to leave the hospital Two events of systemic hypotension occurred, that lead the nurse to pause the ultrafiltration. They are marked by arrows in Fig. 1
Patient related data		Clinical estimated dry weight: 86 kg Endpoint: The patient died after 3 dialysis sessions were conducted

## Discussion and conclusions

This patient case reports a close correlation between fluid balance and mPAP. It is clearly demonstrated that mPAP decreased over time as the dialysis proceeded and the ultrafiltration volume (UF-volume) increased. The curve representing the second dialysis demonstrates an increase in mPAP as the dialysis have been ended, which can be interpreted as return of the 250 ml extracorporeal blood volume. The pressure decreases again at the final post-dialytic measurement, which we interpret as movement of fluid from the intravascular back to interstitial compartment. MAP remained relatively stable during dialysis. We observed that pre-dialytic mPAP was relatively constant at 44, 42, and 41 mmHg in concordance with the patient’s interdialytic weight gain. Thus, we found mPAP to be sensitive towards fluid status, however we cannot safely assume the changes to mPAP to be driven solely by ultrafiltration. The relation between PAP and fluid status is somewhat supported in literature, though the pathophysiology behind PH in ESRD patients is still not fully understood and falls under clinical PH category 5: *PH with unclear and/or multifactorial mechanism, including chronic renal failure* [5]. The PEPPER-trial suggests a great proportion of PH in chronic kidney disease (CKD) patients to be related to fluid status, as they found most of the hemodialysis patients suffering from PH to have post-capillary hypertension [5]. This is usually related to fluid overload and left ventricular dysfunction [8]. The PEPPER-trial found post-dialytic mPAP to be 55 ± 17 mmHg in their dialysis population [5]. In comparison, the post-dialytic mPAP in this patient was 38, 36 and 30 mmHg.

Of interest, the patient of this case developed clinical symptoms in terms of two events of hypotension during dialysis, which lead the nurse to pause the ultrafiltration. Neither of these events were associated with pulmonary hypotension; on the contrary, the corresponding mPAP at these two events where 29 and 32 mmHg, thus well above the cut-off for pulmonary hypertension for the general population. This raises the question of whether the dry weight was set too low or rather if the patient was unable to compensate for the UF-rate as the pressure becomes nearer that recommended for general population.

To compare with existing methods of assessing hydration status, we estimated hydration status by clinical estimated dry weight and bioimpedance measurement.

The patient reached his prescribed dry weight as the post-dialytic weight for dialysis session 3, with a corresponding mPAP of 30 mmHg still hypertensive by definition. This would seemingly suggests that the patient was still overhydrated, thus dry weight overestimated. The highly elevated pro-BNP levels could somewhat support the patient being overhydrated, however bearing in mind that pro-BNP is strongest as a negative predictive parameter [9]. The cutoff value for pro-BNP needs to be modified according to the severity of renal dysfunction, as pro-BNP undergoes renal clearance. A pro-BNP cut-off at 7200 pg/ml (851 pmol/l) has been suggested to discriminate between hemodialysis patients with and without left ventricular dysfunction [9] and our patient’s pro-BNP level of 1120 pmol/l is thus well above.

We found clinical hydration status to correlate better with PAP than bioimpedance: The BIA-assessed OH was negative in the end of all the dialysis, thus indicating underhydration even if the patient remained above the prescribed dry weight and mPAP remained elevated. For the final dialysis session, the pre-dialytic BIA-assessed OH level at − 0.1 l indicated no need of ultrafiltration, even if the corresponding mPAP at 41 mmHg and body weight did. It seems that the overhydration assessed by BIA were underestimated in all measurements, and inaccuracy of BIA has also been reported in some literature [1, 2].

One great limitation to this case report is the limited number of dialysis sessions, which was all that was attainable. This makes it difficult to draw any final conclusions. Keeping this in mind, CardioMEMS seemed superior in assessing hydration status in this patient case, and of interest the patient remained pulmonary hypertensive through intradialytic symptoms and prescribed dry weight, questioning the cutoff value for this anuric patient.

The novelty of the approach of this case report does not allow for many supporting studies. However, a conference abstract on PAP and fluid status supports the findings of this case: The study, based on six patients doing 10–12 dialysis sessions, found a pre-dialytic systolic PAP at 36.6 ± 10.4 mmHg and concluded that the systolic PAP changes during dialysis mainly depends on fluid status [10].

In conclusion, this case report suggests monitoring pulmonary pressure by the CardioMEMS device could hold exciting prospects as a potential real-time guidance for the nephrologist in assessing fluid status and appropriate dry weight in hemodialysis patients. This case of clinical intradialytic symptoms combined with pulmonary hypertension raises the question of what cut-off should be established for pulmonary pressure in anuric dialysis patients. Further studies are needed to confirm the findings of this case, their applicability in assessing optimal fluid balance and investigate what the cut off for PAP in the dialysis population should be.

## Data Availability

All data generated or analysed during this study are included in this published article.

## Abbreviations

PH: Pulmonary hypertension
ESRD: End-stage renal dysfunction
RHC: Right-heart catheterization
PAP: Pulmonary artery pressure
CHF: Congestive heart failure
mPAP: Mean pulmonary artery pressure
MAP: Mean artery pressure (systemic)
BIA: Multi frequency bioimpedance
OH: Overhydration
UF: Ultrafiltration
CKD: Chronic kidney disease
